# Transparent Automated Advice to Mitigate the Impact of Variation in Automation Reliability

**DOI:** 10.1177/00187208231196738

**Published:** 2023-08-27

**Authors:** Isabella Gegoff, Monica Tatasciore, Vanessa Bowden, Jason McCarley, Shayne Loft

**Affiliations:** 2720 The University of Western Australia, Perth, WA, Australia; 2694 Oregon State University, Corvallis, OR, USA; 2720 The University of Western Australia, Perth, WA, Australia

**Keywords:** automation reliability, automation transparency, human-automation teaming, uninhabited vehicle management

## Abstract

**Objective:**

To examine the extent to which increased automation transparency can mitigate the potential negative effects of low and high automation reliability on disuse and misuse of automated advice, and perceived trust in automation.

**Background:**

Automated decision aids that vary in the reliability of their advice are increasingly used in workplaces. Low-reliability automation can increase disuse of automated advice, while high-reliability automation can increase misuse. These effects could be reduced if the rationale underlying automated advice is made more transparent.

**Methods:**

Participants selected the optimal UV to complete missions. The Recommender (automated decision aid) assisted participants by providing advice; however, it was not always reliable. Participants determined whether the Recommender provided accurate information and whether to accept or reject advice. The level of automation transparency (medium, high) and reliability (low: 65%, high: 90%) were manipulated between-subjects.

**Results:**

With high- compared to low-reliability automation, participants made more accurate (correctly accepted advice *and* identified whether information was accurate/inaccurate) and faster decisions, and reported increased trust in automation. Increased transparency led to more accurate and faster decisions, lower subjective workload, and higher usability ratings. It also eliminated the increased automation disuse associated with low-reliability automation. However, transparency did not mitigate the misuse associated with high-reliability automation.

**Conclusion:**

Transparency protected against low-reliability automation disuse, but not against the increased misuse potentially associated with the reduced monitoring and verification of high-reliability automation.

**Application:**

These outcomes can inform the design of transparent automation to improve human-automation teaming under conditions of varied automation reliability.

## INTRODUCTION

Automated decision aids are increasingly prevalent in modern workplaces. For example, in air traffic control, decision aids advise controllers of potential future conflicts (i.e., loss of minimum separation standards) between aircraft (Noskievič & Kraus, 2017), and decision aids are used in Defense to provide advice regarding the management of uninhabited vehicles (UVs; U.S. Air Force, 2015). Automated decision aid advice in such work contexts is often highly reliable, but not perfect, potentially leading to *disuse* of automated advice (rejecting correct advice) or *misuse* of automated advice (accepting incorrect advice) by the human operator (Lee & See, 2004; Parasuraman & Riley, 1997).

### Automation Reliability

Automation reliability is a key factor determining operator reliance on automation and the accuracy of automation use (e.g., Hussein et al., 2020a; Madhavan & Wiegmann, 2007; Metzger & Parasuraman, 2005; Rovira et al., 2007; Shah & Bliss, 2017; Wiegmann et al., 2001). Models of supervisory monitoring that have emerged from engineering disciplines (Senders, 1983; Sheridan, 1970; see Moray, 1986 for review), and related computational models of human control of attention in visual workspaces (Steelman et al., 2011; Wickens et al., 2015), assume that the probability/degree to which humans process information depends on the relative expected value (relevance) and cost of accessing that information (also see Fu & Gray, 2006). Some theorists subsequently argue that it is adaptive to monitor/verify automated advice less if that automation has high historical reliability (Moray, 2003; Moray & Inagaki, 2000). As a consequence of reduced monitoring and verification, high automation reliability can increase the acceptance of automated advice, increase trust in automation, decrease decision time, and reduce subjective workload. However, it can also lead operators to misuse historically highly reliable automated advice, that is, to accept the automation’s rare incorrect advice uncritically (Barg-Walkow & Rogers, 2016; Hussein et al., 2020b; Parasuraman & Riley, 1997; Rovira et al., 2007). Conversely, the aforementioned supervisory monitoring/attention control models predict that low-reliability automation will increase the extent to which operators monitor and verify automated advice, which can increase decision time and subjective workload, and decrease trust and reliance on automation (e.g., Hussein et al., 2020b; Parasuraman & Riley, 1997; Rovira et al., 2007). To the extent the monitoring and verification of automated advice is excessive, this can lead operators to disuse historically low-reliable automated advice (i.e., reject correct automated advice; Lee & See, 2004; Parasuraman & Riley, 1997).

### Automation Transparency

Another factor contributing to the disuse and misuse of automated advice is that operators can face challenges understanding the reasoning process behind automated advice. One solution is to increase *automation transparency* to allow operators to better understand the rationale underlying advice and what will occur if that advice is actioned. Two main theoretical models of transparency have been proposed: Lyons’ (2013) Robot-to-Human Transparency and Chen et al.’s (2014) Situation-Awareness Agent-based Transparency (SAT). For example, the SAT model outlines three levels of transparency: the automation’s goals, intentions, and proposed actions (Level 1), underlying advice reasoning (Level 2), and projected future outcomes and associated uncertainty if advice is followed (Level 3).

Studies have investigated the impact of transparency on automation use across various task domains (see Bhaskara et al., 2020; Van de Merwe et al., 2022 for reviews). Transparency typically improves the accuracy of automation use (i.e., correctly accepting/rejecting advice; e.g., Mercado et al., 2016; Tatasciore et al., 2023), though some studies have found either no benefit (e.g., Guznov et al., 2020; Roth et al., 2020) or less accurate automation use due to a bias toward agreeing with automation (Bhaskara et al., 2021). While increased transparency can add display information, most studies have indicated that it decreases or does not impact decision time (e.g., Skraaning & Jamieson, 2021; but see Stowers et al., 2020), and either reduces or does not increase subjective workload (Göritzlehner et al., 2014; Panganiban et al., 2020; but see Guznov et al.). Similarly, while findings are inconsistent, operator trust in automation and perceived automation usability can increase with increased transparency (e.g., Stowers et al., 2016).

The current study used a UV management task which is broadly representative of other contexts where operators make decisions about quantitative data presented on computer displays. Several prior studies have examined the impact of transparency in UV tasks (e.g., Bhaskara et al., 2021; Loft et al., 2021; Mercado et al., 2016; Stowers et al., 2020; for a review see Bhaskara et al., 2020). Common across these studies is that participants were required to select the optimal UV to complete missions by either accepting the automation-recommended UV or rejecting that advice and choosing an alternative. Although methodological details varied across studies, participants were typically provided with low (automation purpose/intent; e.g., SAT Level 1), medium (reasoning process; e.g., SAT Level 1 + 2), or high (projected outcomes/uncertainty; e.g., SAT Level 1 + 2+3) transparency. Increased transparency can improve the accuracy of automation use in UV management tasks (e.g., Mercado et al., 2016; Stowers et al., 2020; Tatasciore et al., 2023; but see Bhaskara et al., 2021) without costs to decision time or subjective workload (e.g., Bhaskara et al., 2020; Mercado et al., 2016; Stowers et al., 2020).

### Automation Reliability and Transparency

Increased transparency could mitigate the aforementioned negative effects of variation in automation reliability by helping operators to better judge whether automation is providing correct information on a trial-by-trial basis, and therefore whether to accept/reject advice. For example, under conditions in which automation reliability is historically low, increased transparency should facilitate understanding of the rationale underlying advice, improving trust calibration by allowing individuals to make trial-by-trial decisions based not only on historical reliability, but also current trial automation-presented information/capability. As such, increased transparency should counter the increased disuse associated with low-reliability automation. With high-reliability automation, as reviewed, we expect individuals to logically adapt by monitoring/verifying automated advice less, which can increase automation misuse. Increased transparency should increase the probability of the operator correctly rejecting erroneous automated advice via increased understanding of automated advice rationale and improving trial-by-trial trust calibration, potentially reducing the increased misuse associated with historically high-reliability automation. Of course, this outcome assumes some minimal form of monitoring/verification of highly transparent advice under conditions in which automation reliability has been historically high.

To our knowledge, only two prior studies have investigated the relationship between reliability and transparency. Hussein et al. (2020a) had participants perform a simulated swarm-based UV task with varying automation reliability (70%, 90%) and transparency (with, without). Higher reliability increased correct acceptance rates, acceptance of advice without verification, trust, and misuse. Transparency increased the accuracy of automation use and trust, but did not interact with reliability on any outcome measure. Wright et al. (2020) had participants monitor a simulated dismounted soldier team accompanied by an autonomous squad member providing advice at one of two reliability levels (67%, 100%) and transparency. They only found that trust increased with increased reliability.

### The Current Study

We examined the extent to which increased transparency could mitigate the potential negative effects of low and high automation reliability. Outcome variables were the accuracy of automation use, decision time, subjective workload, perceived trust in automation, and perceived usability. Participants were required to select the optimal UV to complete missions based on vehicle capabilities (e.g., discoverability), capability importance weightings, and environmental factors impacting capabilities. The Recommender (decision aid) advised the optimal UV to complete missions as Plan A, and an alternative UV as Plan B. Participants decided whether to choose Plan A or to reject the advice and choose Plan B.

A 2 (transparency) × 2 (automation reliability) between-subjects design was used. Our transparency design was broadly informed by Lyons’ (2013) and Chen et al.’s (2014) models, but also via direct consultation with Australian Defence. Participants were provided either medium (broadly equating to SAT Level 1 + 2) or high (broadly equating to SAT Level 1 + 2+3) transparency. Low transparency (broadly equating to SAT Level 1) was not included as it has consistently resulted in poor outcomes (see Bhaskara et al., 2020 for review). Medium and high transparency provided participants with information regarding how the Recommender evaluated the importance of UV capabilities based on weightings, which UV the Recommender considered to be better/poorer, and a comparison of the Recommender’s final calculated score, for each UV capability. High transparency additionally provided information regarding *how* the Recommender calculated its final scores by including which environmental factors it considered, and the level of projected impact of environmental factors, for each UV capability.

Participants were randomly assigned to either the low (65%) or high (90%) reliability condition. Automation was considered “reliable” when the Recommender provided accurate information *and* advised the correct UV as Plan A. Automation was considered “unreliable” when it provided inaccurate information, regardless of whether it ultimately advised the correct UV as Plan A. The Recommender could provide inaccurate information by either omitting relevant environmental factors from its calculations and/or incorrectly calculating the level of impact of relevant environmental factors on UV capabilities. In real-world settings, automation can be unreliable by providing inaccurate information; however, still advise correct task actions due to other variables at play or contextual contingencies.

Relatedly, participants were required to determine whether the Recommender was providing accurate or inaccurate information, as well as whether they believed the recommended plan was correct. Our rationale was that increased transparency should not only increase the probability of operators correctly using advice, but also help them recognize whether the information provided was accurate or not, thereby demonstrating understanding of *why* they are choosing to accept/reject the advice. This novel design feature allowed us to determine whether participants were making correct *and* informed, automation-guided decisions, which is crucial in safety-critical work contexts where operators are explicitly tasked with verifying automation-generated recommendations. As such, a Hit was defined as both correctly selecting Plan A *and* correctly identifying whether the information provided was accurate or inaccurate. A Correct reject was defined as correctly selecting Plan B (where the recommended Plan A was incorrect and therefore information was inaccurate by default). Following the acceptance/rejection of advice, participants rated their confidence in that decision. Ideally, increased transparency should increase the confidence operators have in correct decisions. The study predictions are summarized in Table 1.

**TABLE 1:** Summary of Predictions Regarding the Impact of Automation Reliability and Transparency on Outcome Measures

Measure	Impact of Reliability	Impact of Transparency	Reliability x Transparency (Expected Interaction)
Accuracy of automation use	Low < high (increased hit rate with high reliability)	Medium < high (increased hit rate with high transparency)	High transparency may mitigate the negative effect of low reliability on hit rate
	Low > high (decreased correct rejection rate with high reliability)	Medium < high (increased correct rejection rate with high transparency)	High transparency may mitigate the negative effect of high reliability on correct rejection rate
Correct decision time	Low > high (decreased decision time with high reliability)	Medium = high (no difference in decision time)	No interaction predicted
Subjective workload	Low > high (decreased subjective workload with high reliability)	Medium = high (no difference in subjective workload)	No interaction predicted
Trust in automation	Low < high (increased trust with high reliability)	Medium < high (increased trust with high transparency)	High transparency may mitigate the negative effect of low reliability on trust
Perceived usability	Low < high (increased perceived usability with high reliability)	Medium < high (increased perceived usability with high transparency)	No interaction predicted
Confidence in correct decisions	Low < high (increased confidence with high reliability)	Medium < high (increased confidence with high transparency)	No interaction predicted

*Note.* Reliability = reliability of automation: low (65%), high (90%); transparency = level of automation transparency (medium, high).

## METHOD

### Participants

Participants were 193 (128 female, 63 male, 2 nonbinary; *M* = 20.6 years) students at The University of Western Australia (UWA) or from the community who participated for course credit or AUD$15 per hour, and a performance incentive (max AUD$18). Participants were randomly assigned to either low reliability + medium transparency (*n* = 48), high reliability + medium transparency (*n* = 49), low reliability + high transparency (*n* = 48), or high reliability + high transparency (*n* = 48) conditions. This research complied with American Psychological Association Code of Ethics and was approved by the UWA Human Research Ethics Office. Informed consent was obtained.

### Uninhabited Vehicle Management Task

The UV task was run on desktop computers each using a single monitor. Participants completed 120 trials (missions) situated on rural, coastal, or urban terrain.

#### Missions

**Figure 1.**
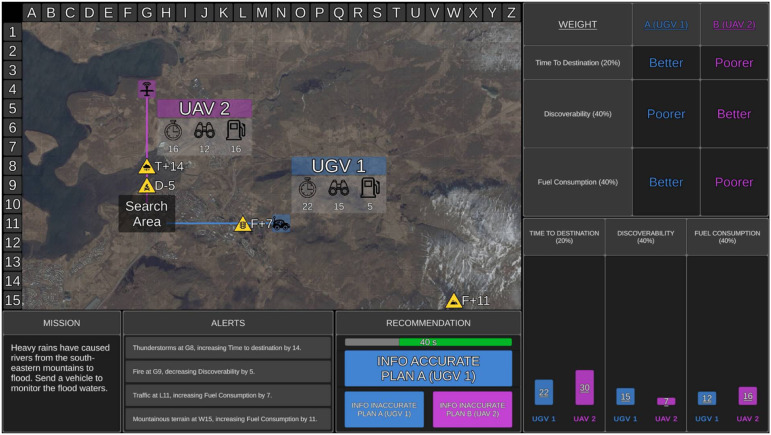
Example of medium transparency. The tactical map presented the two recommended UVs (UGV 1 and UAV 2), their capabilities (time to destination, discoverability, fuel consumption) in a translucent grey box, the path that each UV took to reach the search area (translucent black box), and environmental factors as hazard symbols (three relevant, one irrelevant in this example). The Weighting window presented the importance weightings for each UV capability as percentages (time to destination = 20%, discoverability = 40%, fuel consumption = 40%). The Recommendation window presented the optimal UV as Plan A (UGV 1), the alternative UV as Plan B (UAV 2), and the remaining time available for the mission trial. The table display provided participants with the Recommender’s comparison of the two UVs, labelling each UV as “Better” or “Poorer” for each capability, with larger table rows depicting higher capability weightings (discoverability and fuel consumption are equally weighted for this specific mission). The graphical display showed a visual comparison of the Recommender’s final calculated score for each UV capability, which was represented by the bar graphs. The rural terrain map was used for this mission.

For each trial, participants received a mission statement presented in the Mission window (Figure 1). Mission statements were included for face validity but were not relevant to optimal UV selection. The tactical map showed the location of the search area as a translucent black box. Two UVs were presented on the tactical map and could either be aerial (UAV), ground (UGV), or surface (USV) vehicles. The UVs were randomly numbered 1 or 2 and colored blue or purple. The path that each UV took to the search area was displayed on the tactical map, along with UV capabilities. Time to destination (value presented next to timer symbol in Figure 1) refers to how long the UV would take to reach the search area (lower = quicker). Discoverability (binocular symbol) refers to how discoverable the UV was by third parties (lower = less discoverable). Fuel consumption (fuel gauge symbol) refers to how much fuel the UV would consume to reach the search area (lower = less fuel).

The UV capabilities had different importance weightings on each trial, displayed in the Weighting window. Five weighting combinations were used (Table 2). “Hard” combinations required participants to consider multiple weightings (presented on 60% of trials). For example, the weightings 45%, 45%, 10% required participants to consider either 45% and 45% (combined = 90%) or 45% and 10% (combined = 55%). “Easy” weightings required participants to consider only one large weighting (presented on 40% of trials).

**TABLE 2:** Combinations of Capability Weightings and Their Associated Difficulty Level on Mission Trials

Weighting	Difficulty
45%, 45%, 10%	Hard
40%, 40%, 20%	Hard
40%, 30%, 30%	Hard
60%, 20%, 20%	Easy
80%, 10%, 10%	Easy

*Note.* Hard = need to consider multiple weightings; Easy = need to consider only one large weighting.

Environmental factors were displayed on the tactical map as yellow hazard symbols. Next to each hazard symbol was the letter T (time to destination), D (discoverability), or F (fuel consumption) to indicate which UV capability was impacted, along with a plus or minus sign and level of impact (expressed as a number). Four environmental factors (e.g., thunderstorms) were present on each trial. Participants were informed that relevant factors, indicated by hazard symbols placed directly on a UV path, would impact UV capabilities, whereas irrelevant factor hazard symbols were those not on a UV path. Relevant factors could positively (e.g., decrease discoverability) or negatively (e.g., increase fuel consumption) impact UV capabilities. On each trial, there was one, two, or three relevant factors, or none (i.e., all irrelevant). The four environmental factors on the tactical map, and their associated impact, were also presented as messages in the Alerts window.

The Recommender advised the optimal UV to complete each mission as Plan A and the alternative UV as Plan B, taking into consideration the aforementioned UV capabilities, their relative weightings, and how relevant environmental factors impacted capabilities. The advice was presented in the Recommendation window. Participants were required to decide within 60 s whether to agree with the Recommender and choose Plan A or to choose Plan B. Participants were also required to determine whether the Recommender provided accurate or inaccurate information, as described in further detail below, irrespective of whether they deemed the recommended plan as correct.

After each trial, participants rated how confident they were in their UV selection. They then received feedback on whether the Recommender had advised the correct plan, whether it provided accurate information, whether participants selected the correct plan, whether they correctly identified if the information was accurate, and their decision time.

#### Manipulation of Transparency

**Figure 2.**
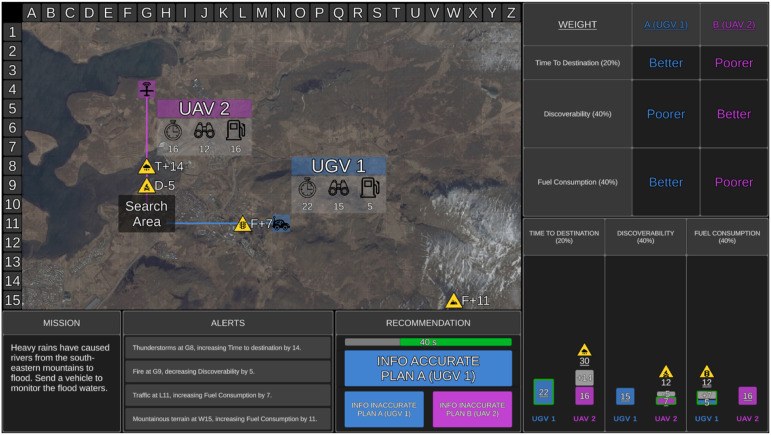
Example of high transparency. The display features were identical to the medium transparency condition (Figure 1), with the following exceptions. The graphical display additionally presented the hazard symbol above each bar in cases where the Recommender considered a relevant environmental factor, and if so, the numerical value that the Recommender either added or subtracted from the value of the final score (the final score was underlined). A green outline also surrounded the bars where a UV performed better on a capability based on the final scores (i.e., UGV 1 for time to destination, UAV 2 for discoverability, UGV 1 for fuel consumption).

Transparency was presented in table and graphical displays (Figures 1 and 2). The table display was identical for both transparency conditions. It showed how the Recommender evaluated UV importance weightings by displaying smaller table rows for lower capability weightings, and larger table rows for higher capability weightings. The table display provided participants with a comparison between which UV was “Better” and “Poorer” on each capability.

The medium transparency graphical display showed three bar graphs, one for each UV capability, allowing direct comparison between the two UVs. Each bar graph contained the Recommender’s calculated score for the corresponding UV capability after considering relevant environmental factors. Shorter bars indicated less time to destination, lower discoverability, and lower fuel consumption (i.e., better performance on a UV capability).

High transparency included the same information as medium transparency, but provided additional information indicating how the Recommender calculated the final score for each UV capability (Figure 2). Relevant hazard symbols were presented above bars on the graph when the Recommender had factored an environmental factor into its calculated UV score. Each bar graph contained the original score for each UV capability and the numerical value that the Recommender added/subtracted from the original score due to a relevant environmental factor (grey portion of the bar). The resulting final score for each UV capability was underlined. When a factor increased the value of the original score, the final score was presented above the bar (see UAV 2 time to destination in Figure 2). When a factor decreased the original score, the final score was presented within the bar (see UAV 2 discoverability in Figure 2). The bar of the UV that the Recommender calculated to be better on each capability was outlined in green.

The Recommender could provide inaccurate information in two ways: it could omit a relevant environmental factor from its calculations entirely, or it could correctly identify a relevant factor, but incorrectly calculate its impact. For example, if time to destination increased by 14-min for UAV 2 due to an environmental factor (Figures 1 and 2), the Recommender may incorrectly increase time to destination by 10-min and provide an inaccurate score for that UV capability. When there were multiple relevant factors, the Recommender could provide inaccurate information on one or more of these factors by either omitting or miscalculating the impact, or a combination of the two errors.

In addition to accepting or rejecting the advised UV plan, participants were required to determine whether the Recommender had provided accurate information. When the Recommender provided accurate information and correctly advised Plan A, participants were instructed to select “Info Accurate Plan A” in the Recommendation window. The consequence of inaccurate information on the Recommender’s advised Plan varied depending on the extent of the error(s). A consequential error occurred when the Recommender provided inaccurate information and advised the incorrect plan. In these cases, participants were instructed to select “Info Inaccurate Plan B.” An *inconsequential* error occurred when the Recommender provided inaccurate information, but the advised Plan A was still correct. In these cases, participants were required to select “Info Inaccurate Plan A.”

In the medium transparency condition, when the Recommender omitted or miscalculated the impact of a relevant environmental factor, the graphical display presented an inaccurate final score, and the table display may inaccurately label a UV capability as “Better” or “Poorer.”

In the high transparency condition, when the Recommender omitted a relevant environmental factor, the hazard symbol was missing from the bar graph, indicating that the relevant factor had not been considered. The number that the Recommender should have added/subtracted for the factor was also missing, therefore presenting an inaccurate score. When the Recommender identified but miscalculated the impact of a factor, the hazard symbol was presented on the bar graph, indicating that the factor had been considered for that UV capability. However, the Recommender could inaccurately add/subtract the value of the factor, resulting in the graphical display presenting an incorrect value that was added or subtracted from the original score, and an inaccurate final score. When the Recommender omitted or miscalculated the impact of a factor, it potentially inaccurately labelled a UV capability as “Better” or “Poorer” in the table display.

#### Manipulation of Reliability

Automation reliability was either 65% (low) or 90% (high); however, participants were not told the exact reliability. Participants in the low-reliability condition were instructed that “The Recommender is only reasonably reliable automation, and as such, it may be relatively common that it may present information inaccurately and/or not recommend the optimal plan.” Participants in the high-reliability condition were instructed that “Although the Recommender is highly reliable automation, it is not perfect, and as such, it may occasionally present information inaccurately and/or not recommend the optimal plan.” Participants in both reliability conditions were instructed that the automation could make errors by missing or miscalculating the impact of environmental factors, or a combination of these errors.

Table 3 shows the proportion of trials that the Recommender provided the correct plan and accurate information. A trial was reliable when the Recommender provided accurate information *and* advised the correct UV as Plan A. A trial was unreliable when the Recommender provided inaccurate information, regardless of whether the advised plan was correct.

**TABLE 3:** Number (%) of Trials Where the Recommender Provided Accurate/Inaccurate Information for the Low and High Reliability Conditions

Correct Advised Plan A	Information Accurate/Inaccurate	Reliability	Low-Reliability Condition	High-Reliability Condition
Yes	Accurate	Reliable	78 (65%)	108 (90%)
Yes	Inaccurate	Unreliable	21 (17.5%)	6 (5%)
No	Inaccurate	Unreliable	21 (17.5%)	6 (5%)

Across the two reliability conditions, 90 (75%) trials were identical (same mission design features and response outcomes) and 20 (16.7%) were identical except for whether the Recommender provided accurate/inaccurate information and advised the correct plan. The remaining 10 (8.3%) trials were unique to ensure a consistent difficulty level between the reliability conditions.

A randomized yoked design was used such that one participant from each of the four conditions received trials in the same randomized order, with only the transparency level and outcome of trials (i.e., whether the correct plan was advised, and whether information was accurate) differing as a function of reliability condition.

### Measures

#### Accuracy of Automation Use and Decision Time

Hit rate was the proportion of trials that participants both correctly selected Plan A and correctly identified whether the information provided was accurate or inaccurate. Correct rejection rate was the proportion of trials that participants correctly selected Plan B (whereby the recommended Plan A was incorrect and therefore information provided was inaccurate by default). Decision time was based on correct decisions only.

#### Workload

Subjective workload was measured using the NASA Task Load Index (Hart & Staveland, 1988). Participants rated their workload from 0 to 100 on six subscales: mental, physical, and temporal demands, performance, effort, and frustration. They then completed 15 pairwise comparisons to indicate which subscales contributed greater to workload. A final score (ranging; 0–100) was calculated by multiplying the rating for each subscale by its weighting, adding the values of subscales, and then dividing the total by 15.

#### Trust

Trust was measured using a modified version of the Merritt (2011) scale. The scale included six items (e.g., “I trust the Recommender”) measured on a 5-point Likert scale, ranging from 1 (strongly disagree) to 5 (strongly agree).

#### System Usability

Perceived usability was assessed using an adapted version of the System Usability Scale (Brooke, 1996). The scale included 10 items (e.g., “I thought the Recommender was easy to use”) measured on a 5-point Likert scale, ranging from 1 (strongly disagree) to 5 (strongly agree). Some items were reversed scored and all items were added together then multiplied by 2.5 to yield a score from 0 to 100.

#### Confidence

Confidence in decisions was assessed after each trial. Participants were asked “How confident are you that your decision to select the UV is correct?” They responded by clicking on a 10-point scale ranging from 1 (“not at all confident”) to 10 (“completely confident”). Confidence in correct decisions was calculated.

### Procedure

Participants watched a 20-min audio-visual training that explained how to perform the task manually, before completing 20 training trials with no automation. This was followed by 20-min condition-specific (medium or high transparency) audio-visual training that explained the Recommender. Participants then completed 120 trials (with a 60 s break midway) in their assigned condition. At the end of the experiment, participants completed workload, trust in automation, and system usability questionnaires in a counterbalanced and yoked order across conditions. The experiment duration was 2.5 hours.

## RESULTS

Data was excluded from three participants who were identified as careless responders (responded to more than 5% of trials in less than 1 s) and from one participant who did not follow task instructions.

### Data Analysis and Statistics

Table 4 presents descriptive statistics and intercorrelations between the dependent measures (collapsed across transparency and reliability). There was a strong positive correlation between trust and perceived usability. Moderate positive correlations were found between hit rate and correct rejection rate, usability, and confidence. There was also a moderate positive correlation between correct rejection rate and confidence, and correct decision time and workload. Moderate negative correlations were found between usability and decision time and workload.

**TABLE 4:** Means, Standard Deviations, and Intercorrelations Between Accuracy of Automation Use (Hit and Correct Rejection), Correct Decision Time, Subjective Workload, Trust, Perceived Usability, and Confidence in Correct Decisions Collapsed Across Transparency and Reliability Conditions

Measure	*M*	*SD*	1	2	3	4	5	6	7
1. Hit rate	.91	.09	—						
2. Correct rejection rate	.69	.26	.46**	—					
3. Correct decision time (s)	15.8	6.00	−.21**	.07	—				
4. Subjective workload	55.3	14.9	−.23**	−.15*	.35**	—			
5. Trust	2.72	.83	.25**	−.06	−.26**	−.19**	—		
6. Usability	62.5	16.3	.41**	.19**	−.42**	−.37**	.53**	—	
7. Confidence	8.21	1.50	.34**	.31**	−.02	−.20**	.00	.28**	—

*Note.* Maximum score for workload = 100, trust = 5, usability = 100, and confidence = 10. ^*^p < .05. ^**^p < .001. Effect sizes are weak = .10, moderate = .30, and strong = .50 (Cohen, 1992).

Descriptive statistics for the dependent measures as a function of transparency and reliability are presented in Table 5. We first ran 2 transparency (medium, high) × 2 reliability (low, high) between-subjects ANOVAs. It was predicted that high transparency would mitigate the disuse of automated advice (i.e., decreased hit rate), and decreased trust, associated with low-reliability automation; and mitigate the misuse of automated advice (i.e., decreased correct rejection rate) associated with high-reliability automation. To test this, we conducted planned orthogonal contrasts examining the change in hit and correct rejection rates, and trust, within medium transparency at low- compared to high-reliability automation, and within high transparency at low- compared to high-reliability automation. Effect sizes were estimated using partial eta squared ( $\eta_{p}^{2}$ ; small = .01, medium = .06, large = .14) for *F*-tests and Cohen’s *d* (small = .20, medium = .50, large = .80) for *t*-tests (Cohen, 1992).

**TABLE 5:** Means (Standard Deviations) for Accuracy of Automation Use (Hit and Correct Rejection), Correct Decision Time, Subjective Workload, Trust, Perceived Usability, and Confidence in Correct Decisions by Transparency and Reliability

	Medium Transparency		High Transparency	
	Low Reliability	High Reliability	Low Reliability	High Reliability
Hit rate	.83 (.12)	.93 (.05)	.93 (.07)	.94 (.05)
Correct rejection rate	.68 (.24)	.67 (.23)	.76 (.19)	.65 (.34)
Correct decision time (s)	18.9 (5.46)	17.0 (5.27)	15.4 (6.04)	11.6 (4.74)
Subjective workload	58.7 (13.0)	56.6 (16.9)	55.5 (13.9)	50.6 (14.5)
Trust	2.29 (.74)	3.09 (.75)	2.31 (.72)	3.19 (.66)
Usability	53.9 (15.3)	66.5 (14.4)	58.6 (17.2)	71.0 (13.0)
Confidence	8.01 (1.53)	8.23 (1.51)	8.18 (1.59)	8.41 (1.39)

We ran a further series of 2 transparency × 2 reliability between-subjects ANOVAs across all dependent measures on the 90 identical trials (same mission design features and response outcomes). We found the same, or on some occasions stronger, effects on the dependent measures as the main analyses reported below which include all trials. This confirms the same effects reported below persisted when only trials with common features across the reliability conditions were analyzed.

### Accuracy of Automation Use

**Figure 3.**
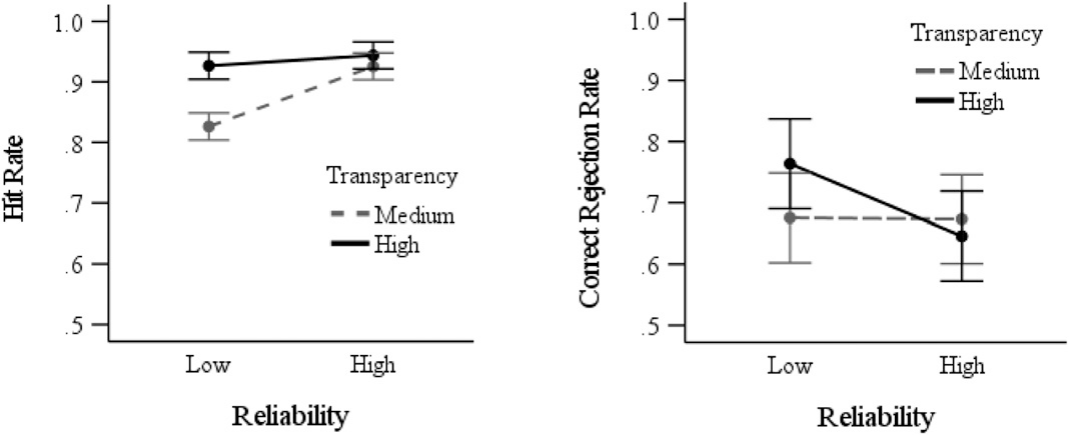
Hit rate (left graph) and correct rejection rate (right graph) as a function of reliability and transparency. Error bars represent 95% between-subjects confidence intervals.

There was a main effect of transparency on hit rate, *F* (1,189) = 27.4, *p* < .001, $\eta_{p}^{2}$ = .13, with hit rates higher with high (*M* = .94, *SD* = .06) compared to medium (*M* = .88, *SD* = .11) transparency. There was also a main effect of reliability, *F* (1,189) = 26.4, *p* < .001, $\eta_{p}^{2}$ = .12, with hit rates higher with high- (*M* = .93, *SD* = .05) compared to low- (*M* = .88, *SD* = .11) reliability automation. These main effects were qualified by an interaction, *F* (1,189) = 13.2, *p* < .001, $\eta_{p}^{2}$ = .07. As seen in Figure 3, medium transparency led to lower hit rates with low- compared to high-reliability automation, *t* (59.6) = 5.17, *p* < .001, *d* = 1.06. In contrast, with high transparency hit rates were not impacted by automation reliability, *t* (94) = 1.41, *p* = .08.

For correct rejection rates, there was no main effect of transparency (*F* < 1), or reliability *F* (1,189) = 2.63, *p* = .11, and no interaction, *F* (1,189) = 2.45, *p* = .12. However, the planned contrasts indicated that in the high transparency conditions, correct rejection rates were lower with high- compared to low-reliability automation, *t* (72.4) = 2.10, *p* = .02, *d* = .43 (Figure 3). Correct rejection rates in the medium transparency conditions were not impacted by automation reliability (*t* < 1).

### Correct Decision Time

There was a main effect of transparency, *F* (1,189) = 33.1, *p* < .001, $\eta_{p}^{2}$ = .15, with faster correct decisions with high (*M* = 13.5, *SD* = 5.73) compared to medium (*M* = 18.0, *SD* = 5.42) transparency. There was also a main effect of reliability, *F* (1,189) = 13.6, *p* < .001, $\eta_{p}^{2}$ = .07, with faster correct decisions with high- (*M* = 14.3, *SD* = 5.69) compared to low- (*M* = 17.2, *SD* = 5.99) reliability automation. There was no interaction, *F* (1,189) = 1.55, *p* = .22.

### Subjective Workload

There was a main effect of transparency, *F* (1,189) = 4.74, *p* = .03, $\eta_{p}^{2}$ = .02, with lower subjective workload with high (*M* = 53.0, *SD* = 14.4) compared to medium (*M* = 57.6, *SD* = 15.1) transparency. There was no main effect of reliability, *F* (1,189) = 2.78, *p* = .10, and no interaction (*F* < 1).

### Trust and Perceived Usability of Automation

#### Trust

**Figure 4.**
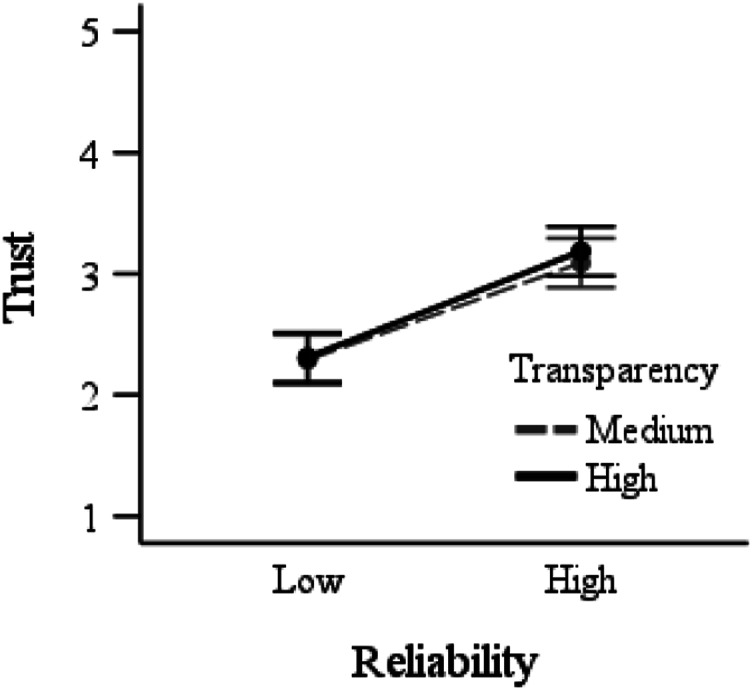
Trust in automation as a function of reliability and transparency. Error bars represent 95% between-subjects confidence intervals.

There was a main effect of reliability, *F* (1,189) = 65.5, *p* < .001, $\eta_{p}^{2}$ = .26, with higher trust with high- (*M* = 3.14, *SD* = .71) compared to low- (*M* = 2.30, *SD* = .73) reliability automation. There was no main effect of transparency, and no interaction (*F*s<1). Planned contrasts indicated that participants trusted high- more than low-reliability automation when provided either medium, *t* (95) = 5.28, *p* < .001, *d* = 1.07, or high, *t* (94) = 6.21, *p* < .001, *d* = 1.27, transparency (Figure 4).

#### Usability

There was a main effect of transparency, *F* (1,189) = 4.56, *p* = .03, $\eta_{p}^{2}$ = .02, with higher usability ratings with high (*M* = 64.8, *SD* = 16.3) compared to medium (*M* = 60.3, *SD* = 16.1) transparency. There was a main effect of reliability, *F* (1,189) = 33.1, *p* < .001, $\eta_{p}^{2}$ = .15, with higher usability ratings with high- (*M* = 68.7, *SD* = 13.8) compared to low- (*M* = 56.3, *SD* = 16.4) reliability automation. There was no interaction (*F* < 1).

### Confidence in Correct Decisions

There was no main effect of transparency (*F* < 1), or reliability, *F* (1,189) = 1.09, *p* = .30, and no interaction (*F* < 1).

## DISCUSSION

We examined the extent to which increased transparency could mitigate the potential negative effects of variation in automation reliability on the accuracy of automation use and trust. We also examined the impact of reliability and transparency on decision time, subjective workload, perceived usability, and confidence. High transparency provided information regarding *how* the Recommender calculated its final UV capability scores, in terms of which environmental factors were considered and their projected impact on UV capabilities. High transparency was expected to allow participants to make more informed judgements regarding whether the automation provided accurate information, and whether to accept or reject automated advice. The predictions and findings are summarized in Table 6.

**TABLE 6:** Summary of Predictions and Findings Regarding the Impact of Automation Reliability and Transparency on Outcome Measures

Measure	Impact of Reliability	Matched Prediction?	Impact of Transparency	Matched Prediction?	Reliability x Transparency (Expected Interaction)	Matched Prediction?
Accuracy of automation use	Low < high (increased hit rate with high reliability)	**Yes**	Medium < high (increased hit rate with high transparency)	**Yes**	High transparency may mitigate the negative effect of low reliability on hit rate	**Yes**
	Low > high (decreased correct rejection rate with high reliability)	No	Medium < high (increased correct rejection rate with high transparency)	No	High transparency may mitigate the negative effect of high reliability on correct rejection rate	No
Correct decision time	Low > high (decreased decision time with high reliability)	**Yes**	Medium = high (no difference in decision time)	No	No interaction predicted	
Subjective workload	Low > high (decreased subjective workload with high reliability)	No	Medium = high (no difference in subjective workload)	No	No interaction predicted	
Trust in automation	Low < high (increased trust with high reliability)	**Yes**	Medium < high (increased trust with high transparency)	No	High transparency may mitigate the negative effect of low reliability on trust	No
Perceived usability	Low < high (increased perceived usability with high reliability)	**Yes**	Medium < high (increased perceived usability with high transparency)	**Yes**	No interaction predicted	
Confidence in correct decisions	Low < high (increased confidence with high reliability)	No	Medium < high (increased confidence with high transparency)	No	No interaction predicted	

*Note.* Reliability = reliability of automation: low (65%), high (90%); transparency = level of automation transparency (medium, high).

### Automation Reliability

Participants used automation more accurately (correctly accepting advice *and* identifying whether information was accurate), and made these decisions faster, when they used high- compared to low-reliability automation. However, they showed no difference in correct rejection rate as a function of reliability. Participants trusted high- more than low-reliability automation, and rated it as more usable. There was no difference in subjective workload or confidence in decisions as a function of reliability.

These findings are generally consistent with prior work (e.g., Hussein et al., 2020a; Metzger & Parasuraman, 2005; Rovira et al., 2007) indicating that high-reliability automation can increase the likelihood of operators accepting correct automated advice, decrease decision time, and increase trust. Contrary to prior findings, at least when the data was collapsed across the transparency manipulation (see further below), we did not find evidence of the misuse of automated advice (i.e., increased acceptance of incorrect advice) typically associated with high-reliability automation use (Barg-Walkow & Rogers, 2016; Parasuraman & Riley, 1997; Rovira et al., 2007). We also did not replicate the common finding in the literature of reduced subjective workload with increased automation reliability (e.g., Metzger & Parasuraman, 2005).

### Automation Transparency

High transparency resulted in more accurate automation use (correctly accepting advice *and* identifying whether information was accurate), faster correct decisions, lower subjective workload, and higher usability ratings, compared to medium transparency. There was no difference in correct rejection rate, trust, or confidence as a function of transparency.

Overall, these findings are generally consistent with prior studies that have examined the impact of transparency in UV tasks (e.g., Loft et al., 2021; Mercado et al., 2016; Tatasciore et al., 2023), and in other task domains (see Van de Merwe et al., 2022). A notable difference is that we found faster decision times and lower workload with increased transparency, contrary to previous work which showed longer decision time (Stowers et al., 2020) or no benefit to workload (Bhaskara et al., 2021; Loft et al., 2021; Mercado et al., 2016; Stowers et al., 2020). This is likely due to our use of icons and graphical visualizations of transparency; whereas other studies have typically used text-based transparency, which may increase the information load placed on operators. Counter to some prior findings, high transparency did not increase trust in automation (see Bhaskara et al., 2020).

### Interaction Between Automation Reliability and Transparency

As expected, under medium transparency, hit rates (correctly accepting advice *and* identifying whether information was accurate) decreased with low-reliability automation. In contrast, under high transparency, hit rates were not affected by reliability. We conclude that higher transparency improved trust calibration by allowing individuals to make trial-by-trial decisions based not only on historical reliability, but also current trial automation-presented information/capability. To our knowledge, this is the first study to demonstrate that increased transparency can mitigate the increased disuse of automated advice (i.e., rejecting correct advice; Lee & See, 2004; Parasuraman & Riley, 1997) associated with using low-reliability automation. Furthermore, we used a strict criterion for what constituted a Hit, thereby demonstrating that increased transparency can not only increase the likelihood of operators correctly using automated advice, but also increase their understanding of *why* they are correctly accepting the advice. This ability is crucial in safety-critical work contexts in which operators need to make correct, but also informed, automation-guided decisions.

Unexpectedly, with medium transparency, correct rejection rates were not affected by reliability. Contrary to our predictions, high transparency did not mitigate against the negative effect of using high- compared to low-reliability automation on the misuse of automated advice (i.e., incorrectly rejecting erroneous advice), with the correct rejection rate declining by 11% when using high- (*M* = .65) compared to low- (*M* = .76) reliability automation. However, it is important to note that with high-reliability, correct rejection rates were still comparable for the high (*M* = .65) compared to medium (*M* = .67) transparency conditions. In addition, the additive main effects of reliability and transparency indicated correct decision times were fastest under high transparency/high reliability conditions.

This combination of findings suggests that participants provided higher transparency did not scrutinize the transparency information as much to verify automated advice when using high- compared to low-reliability automation. These results are predicted by models of supervisory monitoring (e.g., Moray, 1986; Sheridan, 1970) and modern computational models of human control of attention (e.g., Wickens et al., 2015), and are also consistent with increased reliance on highly transparent automation reported under certain conditions in UV (Bhaskara et al., 2021; Tatasciore et al., 2023) and other (e.g., Bussone et al., 2015) task settings. However, based solely on the current data, we cannot determine the extent to which individuals monitored/verified highly transparent automated advice, as compared to accessing but not understanding highly transparent advice. Thus, future research needs to objectively examine how operators allocate attention to increasingly transparent information as a function of varied automation reliability, such as by using eye-tracking metrics or an information masking/uncovering methodology.

### Limitations and Conclusions

The current study used a UV task broadly representative of other settings in which operators use automated advice to make decisions about quantitative data. A limitation however was the use of novice participants that undoubtably differ from experts in motivation, skills, and cognitive ability. Further, in UV operational settings, missions and tasks are more complex and cognitively demanding. Future research should investigate the effects of transparency using higher-fidelity UV tasks (e.g., where operators can propose solutions or adjust the recommended plan based on corrected/updated information). Furthermore, as we did not include a counterbalanced manual (no automation) condition, and as our manual training only presented limited (20) trials, we could not compare automation-aided to unaided performance. It is known that to the extent that trust in automation (or perceived reliability) exceeds the perceived ability to complete a task manually, automation reliance will increase (Gao & Lee, 2006; Hutchinson et al., 2022a, 2022b). Future research should examine the extent to which the current findings hold if automation reliability was markedly lower or higher from human unaided performance.

In conclusion, to our knowledge, this is the first study to demonstrate that increased transparency can mitigate the disuse of automated advice associated with using low-reliability automation. However, high transparency did not mitigate the misuse associated with high-reliability automation, potentially due to reduced monitoring or verification of advice that seems to be inherent under conditions in which reliability is perceived to be high.
